# Genome-wide association analysis of the resistance to infectious hematopoietic necrosis virus in two rainbow trout aquaculture lines confirms oligogenic architecture with several moderate effect quantitative trait loci

**DOI:** 10.3389/fgene.2024.1394656

**Published:** 2024-05-24

**Authors:** Yniv Palti, Roger L. Vallejo, Maureen K. Purcell, Guangtu Gao, Kristy L. Shewbridge, Roseanna L. Long, Christopher Setzke, Breno O. Fragomeni, Hao Cheng, Kyle E. Martin, Kerry A. Naish

**Affiliations:** ^1^ National Center for Cool and Cold Water Aquaculture, USDA-ARS, Kearneysville, WV, United States; ^2^ US Geological Survey, Western Fisheries Research Center, Seattle, WA, United States; ^3^ School of Aquatic and Fishery Sciences, University of Washington, Seattle, WA, United States; ^4^ Department of Animal Science, University of Connecticut, Storrs, CT, United States; ^5^ Department of Animal Science, University of California, Davis, Davis, CA, United States; ^6^ Hendrix Genetics, Sumner, WA, United States

**Keywords:** GWAS-genome-wide association study, IHNV infection, rainbow trout (*oncorhynchus mykiss*), QTL (loci of quantitative traits), aquaculture, heritability

## Abstract

Infectious hematopoietic necrosis (IHN) is a disease of salmonid fish that is caused by the IHN virus (IHNV), which can cause substantial mortality and economic losses in rainbow trout aquaculture and fisheries enhancement hatchery programs. In a previous study on a commercial rainbow trout breeding line that has undergone selection, we found that genetic resistance to IHNV is controlled by the oligogenic inheritance of several moderate and many small effect quantitative trait loci (QTL). Here we used genome wide association analyses in two different commercial aquaculture lines that were naïve to previous exposure to IHNV to determine whether QTL were shared across lines, and to investigate whether there were major effect loci that were still segregating in the naïve lines. A total of 1,859 and 1,768 offspring from two commercial aquaculture strains were phenotyped for resistance to IHNV and genotyped with the rainbow trout Axiom 57K SNP array. Moderate heritability values (0.15–0.25) were estimated. Two statistical methods were used for genome wide association analyses in the two populations. No major QTL were detected despite the naïve status of the two lines. Further, our analyses confirmed an oligogenic architecture for genetic resistance to IHNV in rainbow trout. Overall, 17 QTL with notable effect (≥1.9% of the additive genetic variance) were detected in at least one of the two rainbow trout lines with at least one of the two statistical methods. Five of those QTL were mapped to overlapping or adjacent chromosomal regions in both lines, suggesting that some loci may be shared across commercial lines. Although some of the loci detected in this GWAS merit further investigation to better understand the biological basis of IHNV disease resistance across populations, the overall genetic architecture of IHNV resistance in the two rainbow trout lines suggests that genomic selection may be a more effective strategy for genetic improvement in this trait.

## Introduction

Infectious hematopoietic necrosis virus (IHNV; *Novirhabdovirus salmonid*) is endemic to western North America, and has been spread to parts of Asia, Europe, and the Middle East (Kurath et al., 2003; Enzmann et al., 2005; Adel et al., 2016; Wang et al., 2016). In the western U.S., IHNV causes morbidity and mortality in the commercial rainbow trout (*Oncorhynchus mykiss*) and Atlantic salmon (*Salmo salar*) aquaculture industries, as well as federal, state and tribal salmon and trout hatcheries (Kurath et al., 2003; Troyer and Kurath, 2003; Saksida, 2006; Bootland and Leong, 2009; Breyta et al., 2013). The disease caused by IHNV, infectious hematopoietic necrosis (IHN), is associated with acute mortality with losses as high as 90% (LaPatra, 1998). Rainbow trout fry are the most susceptible life stage but larger fish can still suffer disease (LaPatra et al., 1990). An effective IHNV DNA vaccine exists, but the need to inject the vaccine makes it impractical for immunizing large numbers of rainbow trout fry (Anderson et al., 1996; Lorenzen and LaPatra, 2005). Novel vaccine and delivery methods remain an active area of research, given the importance of IHNV to the trout industry (Plant and LaPatra, 2011; LaPatra et al., 2015; Larragoite et al., 2016). However, at present no cost-effective vaccine is commercially available for rainbow trout fry, and additional approaches are needed to control IHN disease in aquaculture. Genetic improvement for enhanced disease resistance represents such approach. The use of selectively bred resistant animals integrates well into an overall viral disease control strategy that incorporates good animal husbandry, biosecurity, use of specific pathogen free eggs and vaccination if practical, cost-effective and available.

Additive genetic variance is the basis for selective breeding in agricultural animals. Additive genetic variance for resistance to IHNV infection in rainbow trout has been estimated to be moderate based on heritability estimates for IHNV survival status (h^2^ = 0.23–0.55) and survival days (h^2^ = 0.02–0.20) in a steelhead trout (*O. mykiss*) population (Brieuc et al., 2015) and for survival status (h^2^ = 0.25) in the commercial breeding population of Clear Springs Food (CSF) (Vallejo et al., 2019). These results suggest that resistant rainbow trout lines can be developed using family-based selective breeding methods. Furthermore, selective breeding of a rainbow trout line for resistance to IHNV has been implemented at the CSF breeding program since the year 2000 (Campbell et al., 2014), and recently we have shown that genomic-enabled selection can accelerate the improvement of disease resistance in that commercial rainbow trout strain (Vallejo et al., 2020). Here, we examine the opportunity for similar practises in two commercial aquaculture lines that were naïve to previous exposure to IHNV.

Genomic technologies have improved breeding predictions accuracy in agriculture by identifying DNA markers linked to complex phenotypic traits (Barabaschi et al., 2016). Genomic selection (GS) is a selective breeding strategy that examines together the association between all genetic markers genotypes in a population with the trait or traits of interest to predict the breeding value of an individual animal from the population (Meuwissen et al., 2001; Goddard et al., 2011). For example, in rainbow trout aquaculture it was shown that GS can double the accuracy of breeding value predictions for resistance to bacterial cold-water disease (Vallejo et al., 2017a), and in recent years the technology has been widely adopted by the salmonids aquaculture industry and in other aquaculture species (Song et al., 2023; Yáñez et al., 2023). Marker assisted selection (MAS) is a simplified strategy of genomic selection that can be implemented for traits in which most of the genetic variance is controlled a single locus. For example, MAS for infectious pancreatic necrosis (IPN) resistance in Atlantic salmon has resulted in a 75% reduction in the occurrence of that viral disease in the European Atlantic salmon industry (Moen et al., 2007; Houston et al., 2008; Houston et al., 2010; Moen et al., 2015). A key first step in conducting such studies is to identify molecular markers linked to the trait by studying their co-inheritance in segregating populations, using analytical approaches such as genome-wide association studies (GWAS) (Goldstein et al., 2010; Visscher et al., 2012; Fernando et al., 2014; Misztal et al., 2014; Vallejo et al., 2017b; Silva et al., 2019; Vallejo et al., 2019). Such studies are more powerful if they are conducted across populations, because the overall number and overlapping genomic regions identified across populations indicate the potential for genomic selection program and marker assisted selection across different broodstocks. These approaches are particularly effective when attempting to initiate a breeding program for a trait in a population that has not undergone previous selection for that trait.

Troutlodge Inc. maintains a year-round production of eggs though the use of four distinct broodstock populations, with peak spawning in February, May, August and November. The two-year spawning cycle has separated these lines further into even and odd year groups. Phylogenetic analyses based on genotypes from 96 Fluidigm SNP assays (Liu et al., 2016) indicated that all four lines are genetically distinct (Liu et al., 2017). Here, we studied the May 2019 and November 2018 populations because they represent distinct genotypic differences within the breeding program. Identification of conserved regions between these two lines may indicate a higher likelihood of identifying these loci in other commercial trout lines. While these lines have undergone selection for multiple production traits, there has been no intentional selection on IHNV resistance. Therefore, these lines are considered naïve for IHNV and may be polymorphic for genes with large effects on IHNV resistance. The CSF breeding population that has already been subject to IHNV survival selection may be fixed for large effect loci, making it more challenging to identify large effect QTL (Campbell et al., 2014; Vallejo et al., 2019).

Through genome-wide association analyses in the two commercial rainbow trout lines, the objectives of this study were to (1) determine whether the opportunity for genomic selection is consistent across different broodstock lines by comparing the genetic architecture of the trait in the distinct May and November lines; (2) identify whether there were shared genomic regions across lines that are significantly associated with resistance to IHNV, to assess the potential for improving unrelated lines through marker-assisted selection; and (3) identify candidate genes within the QTL regions that will enable future functional studies aimed at identifying the host genes involved in IHNV resistance, which in turn can accelerate genetic improvement through selective breeding and development of more effective treatments or vaccination models.

## Materials and methods

### Ethics statement

All fish work was conducted in accordance with national and international guidelines. The protocol for this study was approved by the Institutional Animal Care and Use Committee of the University of Washington, Seattle, WA (Protocol number 4456-01).

### Fish rearing and IHNV challenge

In February 2019, rainbow trout fry (age of ∼80 days post hatching) from 100 nucleus families from the Troutlodge, Inc. November 2018 spawning population (TLUN 2018) were transported from the Sumner, Washington hatchery to the fish rearing facility of the United States Geological Survey (USGS) Western Fisheries Research Center (WFRC) in Seattle, Washington. The 100 full-sib (FS) families were generated from 100 dams to 62 sires, and they included 37 paternal half-sib (HS) families. The fish were reared at the USGS facility for 90 days until the disease challenge with IHNV. Similarly, in August 2019 fry from 103 nucleus families from the Troutlodge May 2019 spawning population (TLUM 2019) were transported to the WFRC facility and reared there for 30 days prior to the disease challenge. The 103 FS TLUM2019 families were generated using 75 sires and 103 dams, and they included 28 paternal half-sib families.

For the TLUN2018 population, the fish were approximately 5.0 g in size and ∼170 days post hatching at the start of the disease challenge, and ∼21 fish per family were placed in three replicated tanks (final count N = 2,025 total) and immersed in IHNV (strain 220-90) using standard methods (Brieuc et al., 2015). A mock control tank with additional 102 fish was immersed in virus-free cell culture medium and tank water volumes were adjusted to provide a similar biological density as the 3 challenge tanks. IHNV was cultured in the fathead minnow EPC cell line (Winton et al., 2010) at 15°C and viral amount was determined by plaque assay (Batts and Winton, 1989). A pilot trial was conducted to determine optimal dosage that was expected to cause ∼50% mortality over 21 days. Confirmed IHNV free trout fry were immersed in a dose of 2,000 plaque forming units (PFU) per ml in static water with aeration for 1 h. Total water volume in the challenge tanks was kept at ∼153 L with fish density of ∼23 g/L, and for the static immersion challenge the volume was dropped to ∼80 L (∼44 g/L). After 1 h, the water flow was resumed, and the challenge tanks were monitored for moribund fish or mortalities daily for a 21-day period and all survivors were euthanized at 21 days. All experiments were conducted at water temperature of ∼15°C to mimic water temperatures common in the rainbow trout aquaculture industry in Southern Idaho.

For the TLUM2019 population, the fish were approximately 2.0 g in size and ∼110 days post hatching at the start of the disease challenge, and 30 fish per family were placed in three replicated tanks (final count N = 3,049 total) and immersed in IHNV (strain 220-90) water. Challenge conditions were as described above, but the rainbow trout fry were immersed in a dose of 1,000 PFU per ml based on finding of slightly higher susceptibility in the pre-challenge pilot study. Total water volumes were similar to the TLUN2018 population challenge, with a fish density of ∼13 g/L for acclimation and survival trial and ∼25 g/L for the 1-h static immersion challenge.

**FIGURE 1:**
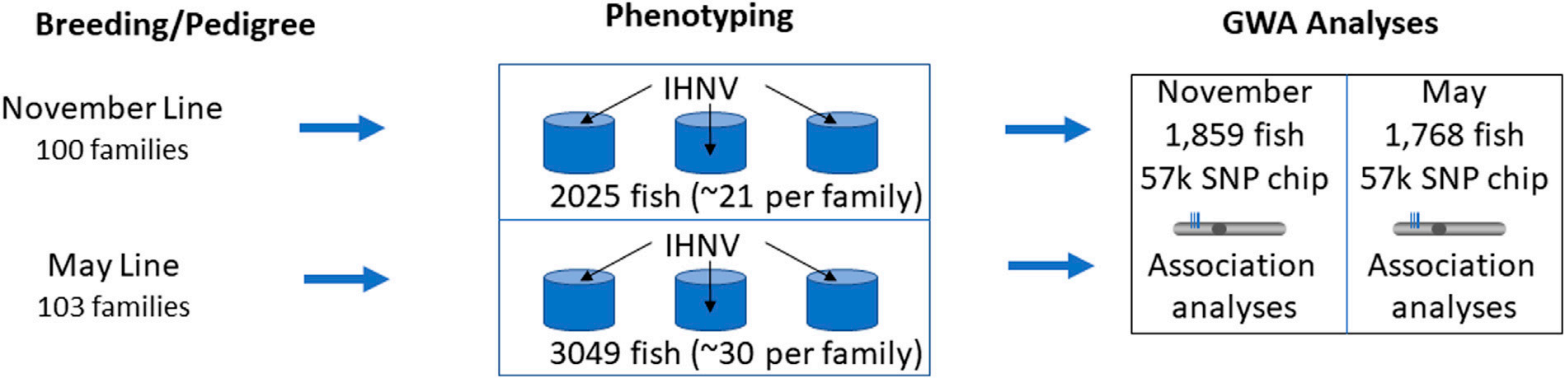
Schematic representation of the experimental design for disease challenges. The Troutlodge November line is represented in this study by year class TLUN2018 and the May line is represented by TLUM 2019.

One main difference in this study compared to Brieuc et al., 2015 was the pooling of individuals from all families and the nearly even distributing the pooled families across three ‘common garden’ replicated tanks. In contrast to individual family tanks where the fish from susceptible families are exposed to higher viral load shedding from their siblings, the ‘common garden’ experimental design has the advantage that all individuals are exposed to the same infection pressure regardless of family. Fin tissues were excised from the parents of the nucleus families at spawning and from all virus-challenged individuals and preserved in 95% ethanol for DNA analysis. After the challenge, identification of an individual to family was performed by genotype analyses. A schematic illustration of the experimental design of the disease challenges is presented in Figure 1.

### IHNV resistance phenotypes

Two phenotypes were recorded; survival days (days to Death; DAYS) and status at the end of the challenge (alive or dead at 21 days; STATUS). However, only the survival days phenotype records were used in the data analyses because survival rate was lower than 10% in the two challenges. Due to the low phenotypic variation in the survival status, and resulting low heritability, this phenotype was excluded from further data analyses.

### Fish populations used for GWAS

TLUN 2018: Eight generations including 7,142 individuals with pedigree records were available for this population. The fish genotyped represent 100 FS families, including 1,859 offspring that were sampled from the three challenge tanks. Due to logistic limitations we had to exclude 150 fish (∼8%) from genotyping. The 50 fish that were excluded from genotyping and pedigree assignments per tank were from the peak mortality days that also represented the median distribution of the mortality per day curve. This exclusion of ∼8% of the fish from the middle of the phenotypic distribution does represent small selective genotyping bias, but we believe that the effect on over estimation of variance components must have been very small.

TLUM 2019: Only two generations of pedigree records (parents and offspring) were available for this population which was used to combine parents from the May spawning odd and even years. The fish genotyped represent 103 full-sib (FS) families, including all the offspring (N = 1,768) that were sampled from the two tanks with the lower mortality rate.

### SNP genotyping and pedigree assignments of offspring

The DNA samples from the two populations were genotyped with the Rainbow Trout Axiom 57K SNP array (Thermo Fisher) following previously described procedures (Vallejo et al., 2019). The samples were genotyped by a commercial service provider (Center for Aquaculture Technologies, San Diego, CA). Following genotype calls, all the SNP markers and samples with call rate (CR) greater than 95% were retained for pedigree assignments. Parentage assignment of individual offspring to all known parental pairs used to produce the full-sib families was conducted using a customized Perl script (Supplementary Material S1). Assignment required at least a 99% Mendelian segregation match between each parent pair and offspring trio.

Before conducting GWAS, the raw marker genotype dataset was filtered using the software BLUPF90 (Misztal et al., 2015). The QC retained samples and SNPs with a genotype calling rate ≥0.97, minor allele frequency ≥0.05, and departures from Hardy-Weinberg equilibrium ≤0.15. Parent-progeny pairs were tested again with the BLUPF90 script for Mendelian error rate (MER). Samples and SNPs were discarded from further analysis if they had MER ≥1% and ≥3%, respectively. Next, we determined the physical map position (Genome Accession GCA_013265735.3) (Gao et al., 2021) of each of the QC filtered markers. A small fraction of the filtered markers (<3%) that were not mapped to chromosome sequences did not have a physical map location, and those markers were excluded from further data analysis. The maps generated for the two populations can be found in Supplementary Material S2, S3.

After genotype data QC and filtering of animals and markers in the TLUN2018 population, a total of 1,796 offspring and 162 parents were used in the association analysis with genotype data from 33,715 informative SNPs. In the TLUM2019 population, 1,742 offspring and 178 parents were successfully genotyped with 35,135 informative SNPs after QC and filtering of animals and markers.

### Estimation of genetic variance parameters

Three methods were used to estimate genetic variance parameters, two of which were based on genetic markers and one used only pedigree and phenotype records.

The DAYS records from TLUN 2018 $\left(n=1796\right)$ and TLUM 2019 $\left(n=1742\right)$ were fit to an animal linear model to estimate genetic variance parameters for survival DAYS in each population dataset, separately. We fitted a linear mixed model for DAYS records using this animal model: $y=1\mu +X_{d}d+\text{Za}+\text{Wc}+e$ , where y is the vector of phenotypic records, 1 is a vector of 1s, μ is the overall mean of phenotypic records, d is a vector of fixed effects, $X_{d}$ is an incidence matrix relating records to fixed effects in d,a is a vector of random individual animal effects, c is a vector of random common environmental effects (i.e., nested effect of families within challenge tank), e is a vector of residual effects, and Z and W are incidence matrices relating records to random animal and random common environmental effects in a and c , respectively.

For the single-step genomic best linear unbiased prediction method (ssGBLUP), the variances of a,c and e are:

$$var\left[\begin{array}{c} a\\ c\\ e \end{array}\right]=\left[\begin{array}{ccc} H\sigma_{a}^{2} & 0 & 0\\ 0 & I\sigma_{c}^{2} & 0\\ 0 & 0 & I\sigma_{e}^{2} \end{array}\right]$$

where $\sigma_{a}^{2}$ , $\sigma_{c}^{2}$ and $\sigma_{e}^{2}$ are additive genetic, common environment and residual variances, respectively, and H is a matrix that combines pedigree ( A ) and genomic ( G ) relationship matrices as in Aguilar et al. (Aguilar et al., 2010), and the variances of a,c and e are estimated by replacing H with A matrix. We used two and three tanks in the TLUM2019 and TLUN2018 populations, respectively, and the effect of tank on DAYS was significant (*p* < 0.05) only in TLUM2019 but not in TLUN 2018. Thus, the effect of tank was included only in the vector of fixed effects $\left(d\right)$ for TLUM 2019.

The genetic parameters were estimated using the pedigree-based BLUP (PBLUP) and PBLUP with genomic data using single-step method (ssGBLUP) under a Bayesian framework, using the software gibbsf90+ from the computer application BLUPF90 (Misztal et al., 2015). Our Gibbs sampling scheme included 250,000 iterations, of which the first 50,000 iterations were discarded; from the remaining 200,000 iterations one sample was saved from every 40 iterations, such that results from 5000 samples were used in the genetic analysis. The proper mixing and convergence of the Markov chain Monte Carlo (MCMC) Gibbs sampling approach was evaluated with the R package CODA (Plummer et al., 2006).

The heritability for DAYS was estimated as: $h^{2}=\sigma_{a}^{2}/\left(\sigma_{a}^{2}+\sigma_{c}^{2}+\sigma_{e}^{2}\right)$ , where $h^{2}$ is the estimated narrow-sense heritability, $\sigma_{a}^{2}$ is additive genetic variance, $\sigma_{c}^{2}$ is variance due to nested effect of families within challenge tank (i.e., common environment effect) and $\sigma_{e}^{2}$ is residual error variance.

We also estimated genetic variance parameters using Bayesian multiple regression with BayesB model (BMR-BayesB) with software JWAS (Cheng et al., 2018) run with option ‘single-step = false’. We fitted a linear mixed model for DAYS records using this animal model: $y=1\mu +X_{d}d+\text{Xb}+\text{Za}+\text{Wc}+e$ , where X is an n×k matrix of observed genotype covariates for k total number of SNPs across the genome for genotyped n individuals, b is a vector of k additive SNP effects, a is a vector of random polygenic effects. The remaining linear model terms were already defined in the analyses based on PBLUP and ssGBLUP, as described earlier. Scaled inverse chi-squared distributions were used for genetic variance and residual variance as described by Fernando et al., 2014; Fernando et al., 2014); in these priors, the degree of freedom was four and the scaled parameters were estimated by assuming that the proportion of variance of the phenotypic data explained by the regression is 0.5.

The BMR-BayesB method fits a mixture model to estimate marker effects, which assumes that there are two types of SNPs: a fraction of SNPs with non-zero effects $\left(1-\pi \right)$ that are drawn from distributions with a marker-specific variance $\left(\sigma_{\alpha }^{2}\right)$ , and another known fraction of SNPs $\left(\pi \right)$ that *a-priori* have zero effect on the quantitative trait (Meuwissen et al., 2001). In our study, the mixture parameter π was assumed to be known and defined to meet the condition k≤n ; where n is the number of fish with genotype records, p is the effective number of SNPs, and $k=\left(1-\pi \right)p$ is the number of markers sampled as having a non-zero effect that are fitted simultaneously in the Bayesian multiple regression model (Garrick and Fernando, 2013). In this study, we used π=0.999 which enabled sampling about 34 and 35 non-zero effect SNPs and fitted in the multiple regression model at each MCMC iteration in the BMR-BayesB analysis performed with TLUN2018 and TLUM2019 datasets, respectively. The MCMC Gibbs sampling scheme and the assessment of correct mixing and convergence of the MCMC iterations were like those described in the section of estimation of genetic parameters with PBLUP and ssGBLUP.

### GWAS with wssGBLUP

The November and May spawning lines are two separate and distinct genetic lines with no shared pedigree. Therefore, the GWAS was performed separately for the two populations using DAYS phenotype and genotype data records from TLUM2019 and TLUN 2018. We conducted GWAS with the wssGBLUP method using 1-Mb sliding SNP windows (Wang et al., 2012; Misztal et al., 2015). Briefly, the effects were calculated for individual SNPs in the first step, as shown below. Afterward, the effects of all SNPs within a 1-Mb distance were added and recorded for each sliding window. Briefly, the 1-Mb window slides by one SNP at a time from the first SNP until the last SNP on each chromosome and the results for SNPs that are included in the window are jointly summarized; thus, the estimates for SNP effects is a moving average of n adjacent SNPs included in the 1-Mb window (Misztal et al., 2015). The choice of a 1-Mb window size was based on our recent estimate of strong LD (*r* ^2^ ≥ 0.25) spanning on average over 1 Mb in the rainbow trout genome (Vallejo et al., 2018).

In GWAS with wssGBLUP, the weights for each SNP are one for the first iteration, which indicates that all SNPs have the same weight (i.e., single-step GBLUP). For the subsequent iterations (2^nd^, 3^rd^, etc.), the weights are SNP-specific variances that are calculated using the estimate of the SNP allele-substitution effect from the previous iteration and the corresponding SNP allele frequencies (Wang et al., 2012). The estimates of SNP effects were calculated using a weighted relationship matrix, using the following equation: $\hat{u}={\mathrm{DM}}^{\prime }{\left[{\mathrm{MDM}}^{\prime }\right]}^{-1}{\hat{a}}_{g}$ , where $\hat{u}$ is the vector of the estimated SNP effects; D is a diagonal matrix of weights for variances of SNP effects; M is a matrix linking genotypes of each SNP to each individual; and ${\hat{a}}_{g}$ is the estimate of the additive genetic effect for genotyped animals. The individual variance of SNP effects, which corresponds to the diagonal elements of D , was estimated as suggested by Zhang et al. (Zhang et al., 2021): ${\hat{\sigma }}_{u,i}^{2}={\hat{u}}_{i}^{2}{2p}_{i}\left(1-p_{i}\right)$ , where: ${\hat{u}}_{i}^{2}$ is the square of the effect at SNP i , and $p_{i}$ is the observed allele frequency for the second allele of SNP i . In this GWAS, we used results from the second iteration of wssGBLUP, because generally they provide the highest accuracy of genomic predictions (Vallejo et al., 2016) and marker effects (Wang et al., 2012; Irano et al., 2016; Melo et al., 2016; Vallejo et al., 2016). The linear model used for GWAS followed that of the ssGBLUP model previously described for the estimation of genetic variance parameters. It was conducted using Gibbs sampling methods implemented in the software gibbsf90+ from the computer application BLUPF90 (Misztal et al., 2015).

### GWAS with bayesian multiple regression

We conducted GWAS for DAYS with BMR-BayesB model using 1-Mb non-overlapping SNP windows (Fernando et al., 2014; Fernando et al., 2016). The BMR-BayesB model uses the same pedigree information and all animals that had phenotype and genotype records, following the wssGBLUP method. The 1-Mb window’s posterior probability of association (WPPA) with the phenotype was used to estimate the window’s proportion of false positive as PFP=1−WPPA (Fernando et al., 2017).

The GWAS for DAYS was performed with BMR-BayesB method (Cheng et al., 2018) implemented in the software JWAS (Cheng et al., 2018). The linear model used for GWAS conformed with the BMR-BayesB model previously described in the section on estimation of genetic variance parameters. The BMR-BayesB uses MCMC Gibbs sampling in the GWAS analysis (Garrick and Fernando, 2013). The MCMC Gibbs sampling scheme and the assessment of correct mixing and convergence of the MCMC iterations have also been previously described (section on estimation of genetic parameters). We did not perform GWAS using sliding SNP windows with BMR-BayesB because this method has not been implemented in the software JWAS.

### Detection of QTL

Quantitative trait loci (QTL) associated with resistance to IHNV were defined as 1-MB SNP windows that explained additive genetic variance (AGV) higher than 1.9% (Vallejo et al., 2022). All QTL windows that were mapped to the same chromosome and were located within less than 20 Mb from each other were defined as belonging to the same QTL region. We have previously used these conservative criteria for defining QTL in GWA analyses in rainbow trout to reduce the type I error rate as much as possible.

For comparison of the QTL genome positions with those previously identified in the Clear Springs Foods (CSF) line (Vallejo et al., 2019), the positions of the flanking SNPs from each QTL in that study were identified on the current version of the rainbow trout reference genome (Gao et al., 2021).

### Identification of protein coding genes in the QTL regions

Standard template queries were used on AquaMine v1.2 (https://aquamine.rnet.missouri.edu/aquamine/) to identify protein coding genes in the most significant QTL SNP windows and to search for the genes description, the GO terms and predicted pathways.

## Results

### IHNV survival phenotypes

**FIGURE 2:**
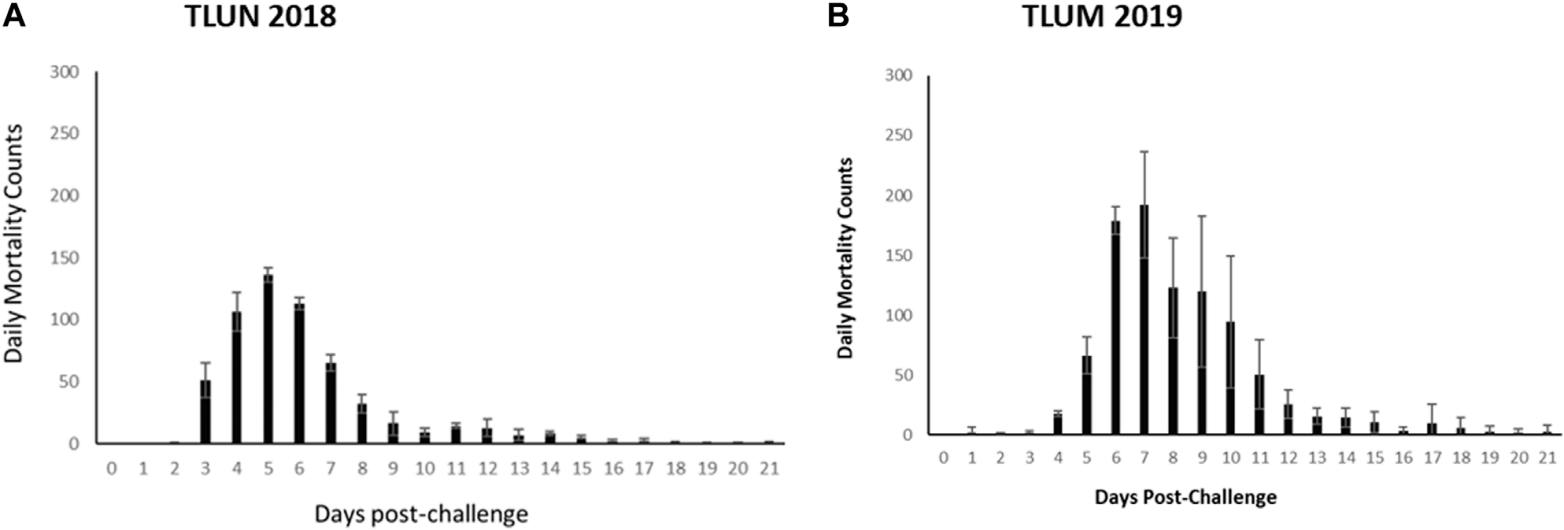
Distribution of daily mortality post IHNV immersion challenge in the commercial rainbow trout TLUN2018 population **(A)** and in the TLUM2019 population **(B)**. The bars represent standard deviation (SD) between tanks.

The mortality rate in both challenges was higher than predicted based on the pilot dosing experiments. In the TLUN2018 population the average mortality per tank was 86% with SD of 2% compared to 2% mortality in the mock trial control tank. In the TLUM2019 population the average mortality per tank was 92% with SD of 11% compared to no mortality in the mock trial. Daily mortality peaked on day 5 in the TLUN2018 challenge and on day 7 in the TLUM2019 challenge (Figure 2).

### Heritability of resistance to IHNV

The estimated heritability for survival days had a range of 0.08–0.25 and 0.15–0.23 across three methods of estimation in TLUM2019 and TLUN 2018, respectively (Table 1). These estimates of low to moderate heritability for the phenotype of survival days underline the potential for genetic improvement of IHNV resistance through selective breeding in these two rainbow trout breeding populations. Due to the very low survival rate in both experiments, heritability for the binary trait of survival on day 21 post challenge was not different from zero (data not shown), and this trait was excluded from genome-wide association analysis.

**TABLE 1:** Genetic parameter estimates for IHNV resistance (survival days, DAYS) in two rainbow trout aquaculture strains.

Strain^a^	Method^b^	Genetic parameter^c^			
		$\sigma_{g}^{2}$	$\sigma_{c}^{2}$	$\sigma_{e}^{2}$	$h^{2}$
TLUM2019	PBLUP	1.97 ± 1.02	1.79 ± 0.53	19.45 ± 0.88	0.08 (±0.04)
	ssGBLUP	3.89 ± 1.04	1.45 ± 0.46	18.34 ± 0.83	0.16 (±0.04)
	BMR-BayesB	5.36 ± 0.76	1.60 ± 0.36	16.47 ± 0.80	0.25 (±0.03)
TLUN2018	PBLUP	8.75 ± 2.17	0.63 ± 0.46	28.21 ± 1.59	0.23 (±0.05)
	ssGBLUP	5.55 ± 1.29	1.01 ± 0.56	30.43 ± 1.21	0.15 (±0.03)
	BMR-BayesB	8.22 ± 1.22	1.86 ± 0.45	27.57 ± 1.36	0.23 (±0.03)

^a^ Variance components analysis was conducted using fish from the year-class (YC) 2019 of the Troutlodge May-spawning strain (TLUM, 2019) and from YC, 2018 of the November-spawning strain (TLUN, 2018).

^b^ Genetic parameters were estimated using pedigree-based BLUP (PBLUP), single-step GBLUP (ssGBLUP), and Bayesian multi regression with Bayes B (BMR-BayesB) methods. The survival days records were analyzed using animal linear models.

^c^ Genetic parameter estimate (± standard error): $\sigma_{g}^{2}$ is the additive genetic variance; $\sigma_{c}^{2}$ is the variance due to the nested effects of families within challenge tanks; $\sigma_{e}^{2}$ is the residual error variance; and $h^{2}$ is the estimate of narrow-sense heritability for survival days.

### QTL associated with resistance to IHNV

**FIGURE 3:**
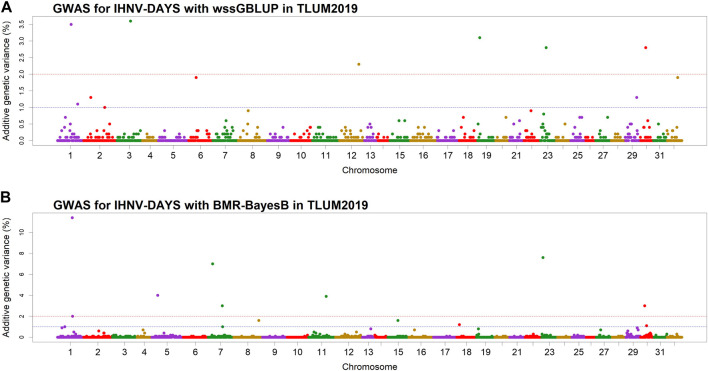
Manhattan plots showing the association of 1-Mb SNP windows with resistance to IHNV in year-class 2019 families from the May-spawning nucleus breeding population (TLUM) using two GWAS models: **(A)** Weighted singe-step GBLUP (wssGBLUP); and **(B)** Bayesian multiple regression with BayesB (BMR-BayesB).

In the TLUM2019 population GWAS, we detected 14 SNP windows of 1-Mb that were associated with resistance to IHNV (AGV≥1.9%) with either the wssGBLUP or the BMR-BayesB GWAS methods (Table 2). The 14 QTL windows were determined to be located within 11 chromosomal regions. Eight of those QTL windows were detected with wssGBLUP and six were detected with BMR-BayesB. Jointly, the 11 QTL regions explained up to 49.7% of the additive genetic variance when accounting only for the largest effect SNPs window detected in each QTL region. As shown in Figure 3, the QTL windows on chromosomes 1 (AGV = 11.4%), 7 (AGV = 7.0%) and 23 (AGV = 7.6%) had the highest proportions of explained additive genetic variance. However, only one QTL window, the window on chromosome 1, had a false positive probability (PFP) lower than 0.05.

**TABLE 2:** Characteristics of quantitative trait loci associated with resistance to IHNV in the TLUM2019 aquaculture strain of rainbow trout^a^.

Chr^b^	AGV (%)^c^	PFP^d^	GWAS method^e^	Physical map (bp)^f^		Window flanking SNPs		SNPs per window
				Start	End	Start	End	
1	11.4	0.03	BMR-BayesB	48,049,099	48,967,892	Affx-88955618	Affx-88930703	17
1	3.5	N/A	wssGBLUP	48,632,135	49,623,888	Affx-88952382	Affx-88938488	24
3	3.6	N/A	wssGBLUP	51,430,431	52,429,704	Affx-88933807	Affx-88905010	25
5	4.0	0.38	BMR-BayesB	19,009,057	19,953,516	Affx-88906315	Affx-88940046	23
6	1.9	N/A	wssGBLUP	25,083,663	26,066,808	Affx-88919141	Affx-88953291	29
7	7.0	0.26	BMR-BayesB	21,017,550	21,868,247	Affx-88941998	Affx-88958253	23
11	3.9	0.44	BMR-BayesB	57,030,022	57,990,833	Affx-88911746	Affx-88928288	20
12	2.3	N/A	wssGBLUP	66,510,299	67,487,681	Affx-88913164	Affx-88923715	27
19	3.1	N/A	wssGBLUP	15,012,572	15,954,248	Affx-88940815	Affx-88911258	15
23	7.6	0.09	BMR-BayesB	13,029,157	13,930,763	Affx-88914566	Affx-88906601	23
23	2.8	N/A	wssGBLUP	28,704,800	29,704,625	Affx-88958162	Affx-88907862	25
30	3.0	0.54	BMR-BayesB	14,136,821	14,976,738	Affx-88904384	Affx-88943429	8
30	2.8	N/A	wssGBLUP	20,284,236	21,267,747	Affx-88907684	Affx-88904888	27
32	1.9	N/A	wssGBLUP	33,372,622	34,329,512	Affx-88926444	Affx-88945805	31

^a^ GWAS, performed using fish from year-class 2019 families from the Troutlodge May-spawning nucleus breeding population.

^b^ Chromosome numbers are based on the USDA_OmykA_1.1 genome assembly (Gao et al., 2021; GenBank Assembly Accession GCA_013265735.3).

^c^ Explained additive genetic variance (AGV) by tested 1-Mb window (%). The 1-Mb windows with AGV≥1.9% were defined as associated with IHNV, resistance.

^d^ PFP is the probability of false positives defined as $PFP=\left(1-WPPA\right)$ , where WPPA is the window posterior probability of association. WPPA was estimated only with the BMR-BayesB model.

^e^ GWAS, conducted using weighted single-step GBLUP (wssGBLUP) and Bayesian multiple regression with BayesB (BMR-BayesB) methods.

^f^ SNP, positions in base pairs (bp) based on rainbow trout reference genome sequence (Gao et al., 2021; GenBank Assembly Accession GCA_013265735.3).

**FIGURE 4:**
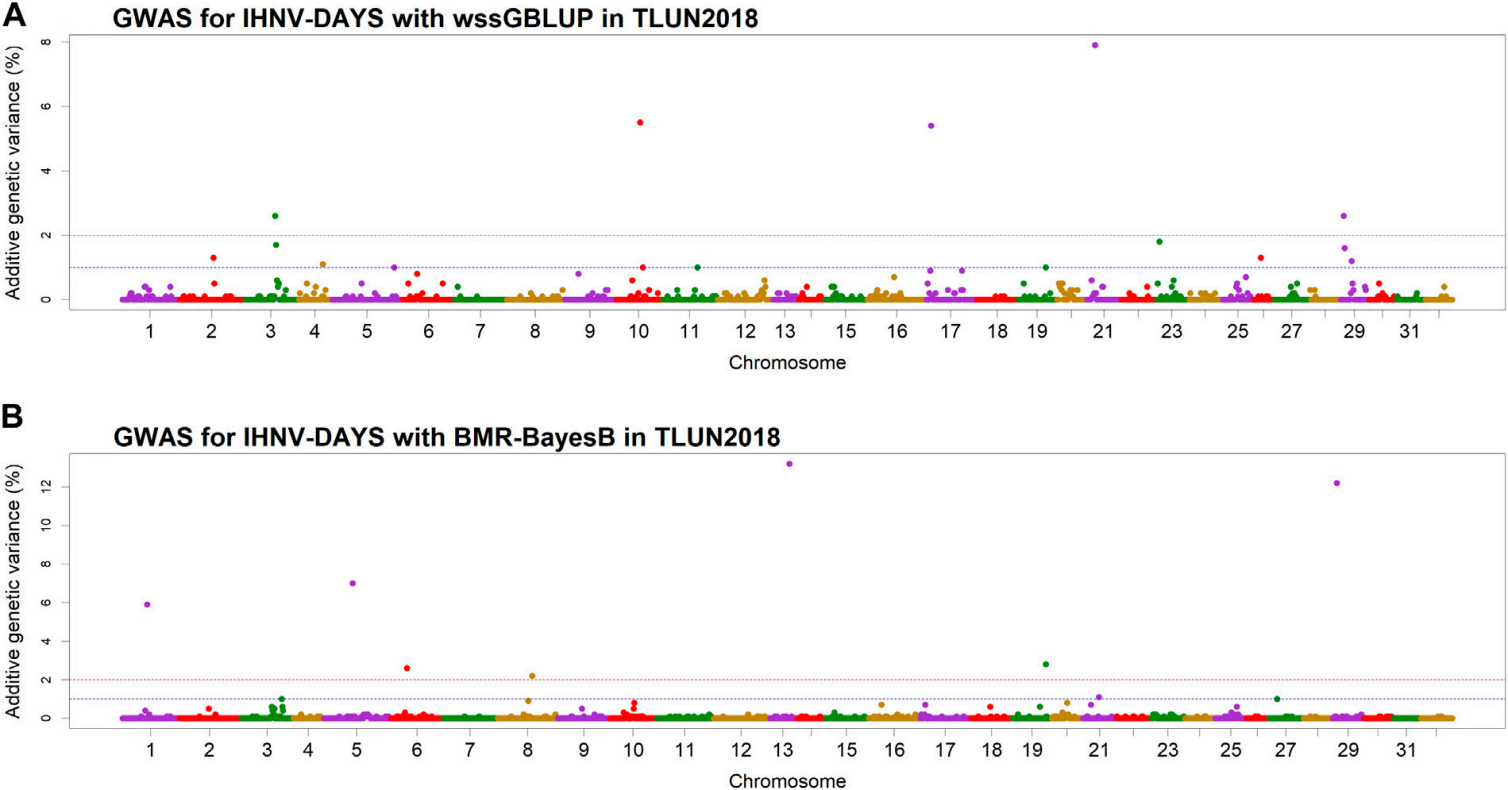
Manhattan plots showing the association of 1-Mb SNP windows with resistance to IHNV in year-class 2018 families from the November-spawning nucleus breeding population (TLUN) using two GWAS models: **(A)** Weighted singe-step GBLUP (wssGBLUP); and **(B)** Bayesian multiple regression with BayesB (BMR-BayesB).

In the TLUN18 population, we detected 13 SNP windows of 1-Mb in 12 chromosomal regions that were associated with resistance to IHNV (Table 3). From these windows, six were detected with wssGBLUP and seven were detected with BMR-BayesB. The 12 QTL regions jointly explained up to 69.23% of the additive genetic variance when accounting only for the largest effect SNP window detected in each QTL region. As shown in Figure 4, the QTL windows on chromosomes 13 (AGV = 13.2%), 21 (AGV = 7.9%) and 29 (AGV = 12.2%) had the highest proportions of explained additive genetic variance. The windows detected with BMR-BayesB on chromosomes 13 and 29 had a false positive probability (PFP) lower than 0.05.

**TABLE 3:** Characteristics of quantitative trait loci associated with resistance to IHNV in the TLUN2018 aquaculture strain of rainbow trout^a^.

Chr^b^	AGV (%)^c^	PFP^d^	GWAS method^e^	Physical map (bp)^f^		Window flanking SNP		SNPs per window
				Start	End	Start	End	
1	5.9	0.34	BMR-BayesB	37,024,181	37,985,996	Affx-88940706	Affx-88919761	19
3	2.6	N/A	wssGBLUP	51,104,028	52,087,029	Affx-88955207	Affx-88951474	26
5	7.0	0.23	BMR-BayesB	43,002,688	43,987,163	Affx-88954192	Affx-88917739	19
6	2.6	0.57	BMR-BayesB	25,050,389	25,985,630	Affx-88944756	Affx-88920694	31
8	2.2	0.68	BMR-BayesB	53,052,503	53,969,345	Affx-88937384	Affx-88925776	28
10	5.5	N/A	wssGBLUP	35,810,116	36,734,512	Affx-88928023	Affx-88930040	40
13	13.2	0.02	BMR-BayesB	41,096,300	41,961,648	Affx-88930116	Affx-88942374	16
17	5.4	N/A	wssGBLUP	26,575,732	27,437,130	Affx-88955005	Affx-88913439	21
19	2.8	0.63	BMR-BayesB	59,071,937	59,743,776	Affx-88919240	Affx-88958570	17
21	7.9	N/A	wssGBLUP	25,853,803	26,848,204	Affx-88919092	Affx-88925348	34
23	1.9	N/A	wssGBLUP	15,680,409	16,648,321	Affx-88911729	Affx-88934547	25
29 (Y)	2.6	N/A	wssGBLUP	4,884,989	5,764,531	Affx-88912960	Affx-88948694	13
29 (Y)	12.2	0.03	BMR-BayesB	6,021,854	6,963,833	Affx-88953281	Affx-88950556	11

^a^ GWAS, performed using fish from year-class 2018 families from the Troutlodge November-spawning nucleus breeding population.

^b^ Chromosome numbers are based on the USDA_OmykA_1.1 genome assembly (Gao et al., 2021; GenBank Assembly Accession GCA_013265735.3).

^c^ Explained additive genetic variance (AGV) by tested 1-Mb window (%). The 1-Mb windows with AGV≥1.9% were defined as associated with IHNV, resistance.

^d^ PFP is the probability of false positives defined as $PFP=\left(1-WPPA\right)$ , where WPPA is the window posterior probability of association. WPPA was estimated only with BMR-BayesB model.

^e^ GWAS, conducted using weighted single-step GBLUP (wssGBLUP) and Bayesian multiple regression with BayesB (BMR-BayesB) methods.

^f^ SNP, positions in base pairs (bp) based on rainbow trout reference genome sequence (Gao et al., 2021; GenBank Assembly Accession GCA_013265735.3).

Two 1-Mb QTL windows were detected within chromosomes 1, 23 and 30 in the TLUM2019 population (Table 2) and chromosome 29 in the TLUN2018 population (Table 3). In each of those four cases we defined the two windows to be part of the same QTL region since those pair of windows were detected within a region smaller than 20 Mb. It is also important to note that within each of those four pairs of 1-MB QTL windows each window was detected by a different GWAS detection algorithm, which further support the findings of true QTL in those four chromosomal regions.

### Predicted protein coding genes identified in QTL regions

Gene content from the most significant QTL was identified by exploring protein coding genes that were annotated by the NCBI Refseq and by the EBI Ensembl. We confined our analyses to the QTL windows that were detected in TLUM2019 on chromosomes 1 (PFP<0.05) and 23 (detected by two GWAS methods and PFP<0.1), and in TLUN2018 on chromosomes 13 (PFP<0.05), 21 (strongest QTL detected with the wssGBLUP method) and chromosome Y or 29 (PFP<0.05). The list of annotated genes from those five QTL regions is provided in Supplementary Material S4, including the start and end positions on the chromosome, and where available, the gene description and the metabolic pathways that were linked to those genes in Refseq. The list of all the Gene Ontology (GO) terms that were assigned to the genes from each of the five QTL regions is presented in Supplementary Material S5.

## Discussion

Overall, we identified oligogenic structure for resistance to IHNV in the two commercial rainbow trout breeding populations that were evaluated in this study, with several moderate-effect QTLs accounting for large portion of the total additive genetic variance (AGV). In the May line (TLUM 2019) we detected 11 QTL regions that jointly explained nearly 50% of the AGV, and in the November line (TLUN 2018) we detected 12 QTL regions that jointly explained nearly 70% of the AGV. Only the QTL 1-Mb windows detected in TLUN2018 on chromosome 21 (25.8-26.8 Mb) and TLUM2019 on chromosome 30 (14.1-14.9 Mb) in this study are co-localized near QTL regions that were detected in previously in a third aquaculture breeding population, in which selection for resistance has occurred (Supplementary Material S6) (Vallejo et al., 2019). Due to the lack of major QTL for this trait in the commercial rainbow trout populations that were analysed, it is likely that genomic enabled selection, rather than marker assisted selection, is the more effective approach for improving the accuracy of breeding predictions for IHNV disease resistance. A similar conclusion was reached in our previous study on the third aquaculture breeding population (Vallejo et al., 2020). Therefore, our hypothesis that a major QTL for resistance to IHNV may be found segregating in the two aquaculture breeding populations - since they do not have history of exposure or selective breeding for IHNV - has not been supported by the results of this study.

**FIGURE 5:**
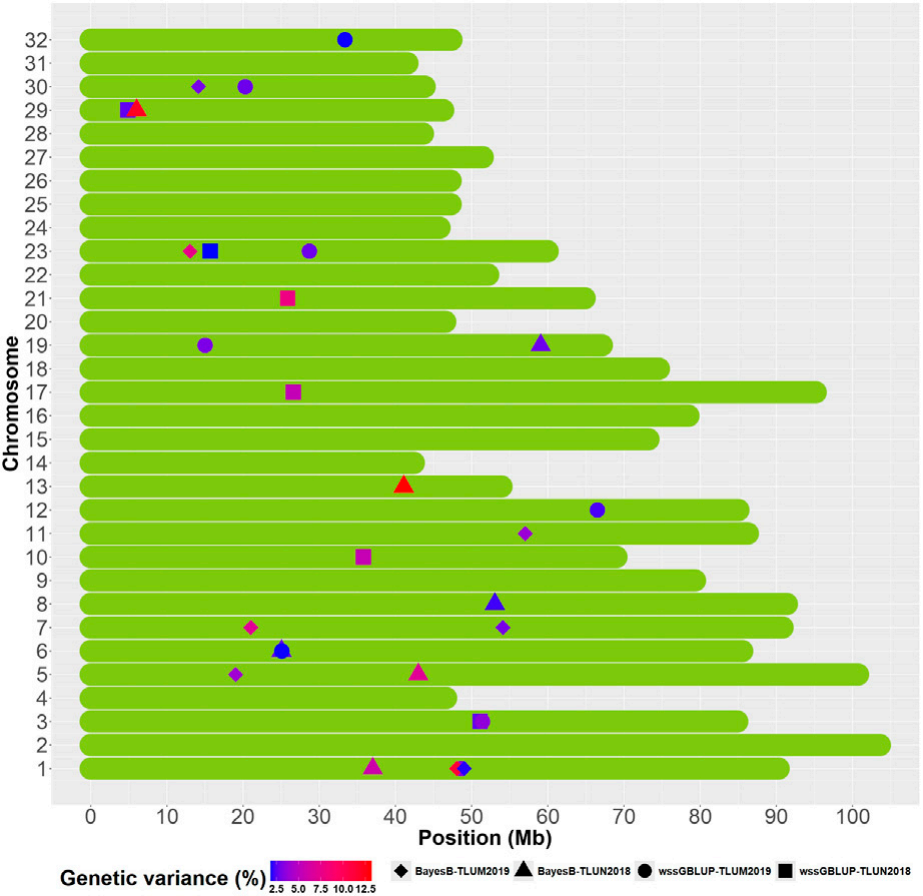
Schematic illustration of chromosome positions of co-localized and unique QTL windows associated with resistance to IHNV (AGV ≥1.9%) in the rainbow trout November 2018 and May 2019 spawning nucleus breeding populations.

We identified overlapping QTL regions located on chromosomes 3 and 6 between the two populations and neighboring QTL regions that may be in linkage disequilibrium (LD) with the same causative gene or genes on chromosomes 1, 5 and 23 (Figure 5). Rainbow trout aquaculture strains have been shown to have long-range LD, particularly on chromosome 5 (Vallejo et al., 2018; Pearse et al., 2019; Vallejo et al., 2019), and thus we cannot rule out the possibility that QTL windows that are even within 20 Mb from each other are in LD with the same causative gene or genes. QTL that are detected in multiple populations are more likely to be true QTL and are also of greater interest for further investigation. The QTL from chromosomes 1 and 23 were also the most significant QTL that were detected in TLUM 2019, making them strong candidates for further evaluation and analyses. Seven QTL located on chromosomes 8, 10, 13, 17, 19, 21 and 29 were unique to the TLUN2018 population and six QTL located on chromosomes 7, 11, 12, 19, 30 and 32 were unique to TLUM 2019. Interestingly, the most significant QTL detected in TLUN2018 from chromosomes 13, 21 and 29 were not shared with TLUM 2019. In comparison with the Clear Springs Food (CSF) population QTL detected in our previous study (Vallejo et al., 2019) we determined that the TLUN2018 QTL on chromosomes 21 and the TLUM2019 QTL on chromosome 30 are co-localized with the CSF QTL that were detected on the same chromosomes (Supplementary Material S6), making QTL-21 that was also one of the strongest QTL in the TLUN2018 population another top candidate for further evaluation.

The age and size of the fish in the two disease challenge experiments was substantially different. The fish from the TLUN2018 population were challenged at the age of ∼170 days post hatching (dph) with an average weight of 5 g and from the TLUM2019 population at the age of ∼110 dph with an average weight of 2 g. Rainbow trout were previously found to remain susceptible to IHNV over a range of sizes but show decreasing susceptibility with increasing size (LaPatra et al., 1990; LaPatra, 1998). For example, rainbow trout ranging from 1.7, 3.4 and 7.4 g exposed to IHNV (1 × 10^5^ pfu/mL) had cumulative percent mortality of 55%, 44% and 18%, respectively (LaPatra, 1998). Previous studies implicate defence barriers or early innate responses as being important for genetic differences in IHNV susceptibility (Quillet et al., 2007; Purcell et al., 2010; Bledsoe et al., 2022). Adaptive immune response such as neutralizing antibodies is important for long-term immunity to IHNV but is unlikely to contribute to survival of naïve fish in an acute challenge (Lorenzen and Lapatra, 1999). Given that the mechanism underlying the impact of age and size on acute IHNV susceptibility has not been characterized, we cannot rule out differential immune responses. However, IHNV susceptibility at two different sizes was found to be a stable trait in both clonal rainbow trout lines (Quillet et al., 2007) and full-sibling rainbow trout families (Purcell et al., 2010). Therefore, while overall susceptibility may change with size, the line or family susceptibility was not found in previous studies to have a transitory effect related to size or age. Taking all this information together, we cannot rule out that the meaningful difference in age and size had an impact on the trait of resistance to IHNV between the two disease challenges in this study and might have contributed in part to the detection of different QTL between the two populations.

The heritability estimates for IHNV resistance in our study were of low to moderate magnitude (0.08-0.25) and somewhat lower than those reported previously in two different rainbow trout population (Brieuc et al., 2015; Vallejo et al., 2019). The different genetic background of those populations may be a factor in the differences in heritability estimates. In addition, one notable difference from those two previous studies was that here we used a common garden design for the disease challenge compared to individual family tanks that were used in the previous studies. Hence, in the current study design the effect of the common shared environment on the family-based response to IHNV infection and survival was reduced, which might have contributed to less inflated heritability estimates compared to the previous studies. Still, the estimated low-moderate heritability in the current study underlines the potential for genetic improvement through selective breeding for IHNV resistance in the two commercial rainbow trout populations.

As described in the methods section, 150 fish (∼8%) were excluded from genotyping in the TLUN2018 population. This exclusion of ∼8% of the fish from the middle of the phenotypic distribution does represent small selective genotyping bias, but we believe that the effect of that small bias on over estimation of variance components must have been very small.

The heritability estimates based on genotype data were comparable across populations when using the same algorithm (0.15–0.16 with ssGBLUP and 0.23–0.25 with BMR-BayesB). Clearly the estimates with BMR-BayesB were higher than ssGBLUP. The difference is caused by the way that the two algorithms estimate the additive genetic variance. In ssGBLUP, the additive genetic variance is calculated using the estimated random individual animal effects. In comparison, with BMR-BayesB the estimate of additive genetic variance is the sum of the additive genetic variance due to random animal polygenic effects (i.e., breeding values of animals) and random marker effects (Fernando et al., 2016).

A larger difference in heritability values was observed using the pedigree and phenotype records (PBLUP; 0.08 and 0.23 for TLUM2019 and TLUN 2018, respectively). This difference may be caused by the reference population that is used by the different models. The reference for the genomic model is the same-generation population from which the genotypes and phenotypes were obtained (also called the training population for genomic selection models), whereas for PBLUP it is the initial generation in the pedigree records (Vallejo et al., 2021; Vallejo et al., 2022). The pedigree records for TLUN2018 trace back through eight generations to a single base population, providing a large dataset of pedigree records to estimate the genetic relatedness of the offspring with phenotypes from this population. In contrast, the TLUM2019 population was formed from a merger of two year-classes that were established from two different base-populations. Thus, the pedigree relies on only one generation of parents and offspring to calculate relatedness among the offspring. Therefore, we believe that the PBLUP heritability estimate for TLUN2018 is more reliable than that for TLUM 2019. The true heritability values for the two populations are likely within the genomic-based estimates of 0.15–0.25.

Two multiple regression GWAS methods were used to identify QTL associated with resistance to IHNV in this study. Such methods are powerful at the population level since they account for linkage disequilibrium between neighbouring loci, and utilize information from all available pedigree, genotype, and phenotype data (Garrick and Fernando, 2013; Misztal et al., 2014). We have previously shown that using two high quality and widely used algorithms for GWAS reveals more information about the genetic architecture of complex disease resistance traits in rainbow trout (Vallejo et al., 2017b; Vallejo et al., 2019; Vallejo et al., 2022). The detection of QTL with both algorithms increases the confidence in the detection of true QTL. In the current study, the QTL on chromosomes 1, 23 and 30 in TLUM2019 and on chromosome 29 in TLUN2018 were detected by both algorithms using the threshold of AGV >1.9. GWAS methods that are based on windows are sensitive to the SNP density and allele frequency (Li et al., 2021). In this study we used 1 Mb windows based on our experience with rainbow trout aquaculture populations where 1 Mb is approximately the average size of the LD extent of *r* ^2^ > 0.25 (Vallejo et al., 2018; Vallejo et al., 2020). We agree that very high or very low number of SNPs per window might affect the signal intensity and that LD is not evenly distributed across the genome.

Several of the genes located within the detected QTL regions may be implicated to be involved in immune response to infection based on their GO term and metabolic pathways predictions. However, it is important to caution that at this point we do not know if the true causative variant for each QTL is found in a protein coding gene. Therefore, our discussion here is limited to the gene functions that are predicted by the gene models that are based on orthology and sequence similarity to genes from model species. Furthermore, for practical reasons we restricted the list of annotated genes to those that were found within the 1-Mb QTL window boundaries, although it is possible that due to linkage disequilibrium with the causative genome variant, the causative genes or sequence variants may be located near rather than inside the SNP windows that were found to have the strongest association with the survival days phenotype. Here, we explore the genes from the five most supported QTL regions that may be involved in the innate or adaptive antiviral immunity of the rainbow trout based on the genome annotation and gene prediction models used in Refseq and Ensembl.

The shiftless antiviral inhibitor of ribosomal frameshifting (shfl) gene is located in the QTL region on chromosome 13. It is an interferon-stimulated gene that in human has been shown to function as a broad-spectrum antiviral factor with suppressive activity against various types of RNA and DNA viruses (Wang et al., 2019; Suzuki and Murakawa, 2022). Also located within the chromosome 13 QTL region is the notch receptor 3 gene (notch3), which is involved in the notch signalling pathway. Among its multiple functions, the notch signalling pathway was also implicated to be involved in innate lymphoid cell fate decision and immune response (Golub, 2021). IL15 receptor alpha chain (il15ra) is located within the QTL region on chromosome 21. The interleukin-15-mediated signalling pathway is involved in STAT activation of natural killing (NK) cells and has been implicated in the suppression of HIV latency (Macedo et al., 2022). Also in this QTL region, suppressor of cytokine signalling 2 (socs2) is a negative regulator of receptor signalling pathway via JAK-STAT and of type I interferon-mediated signalling pathway (Zhang et al., 2021; Kausar et al., 2022). Hence, by their known role in feed-back regulation of signalling pathways associated with viral suppression, both il15ra and socs2 are possible positional candidates for being the causative gene or genes responsible for the association of this chromosomal region with IHNV resistance in rainbow trout. The IKAROS family zinc finger one (ikzf1) is found in the QTL on chromosome 23. The structure and expression pattern of this gene confirm that it is a master switch of hematopoiesis and a key regulator of gene expression in lymphoid cells in rainbow trout (Hansen et al., 1997). In mammals, ikzf1 is required for B-cell development, and has been shown to regulate the development and function of dendritic cells and monocytes in human (Cytlak et al., 2018). Also located in this QTL region is the B cell linker protein, which is a molecular scaffold essential for the B cell receptor signalling pathway and required to promote B cell development (Pappu et al., 1999). The wingless-type MMTV integration site family member 2 (wnt2) is located in the QTL region on chromosome one and in cattle it was found through linkage disequilibrium analysis to be associated with resistance to a pathogenic bacterium and antibody response (Pauciullo et al., 2015). The QTL on chromosome Y (29) does not contain genes that can be implicated directly in antiviral immunity based on their functional annotation. However, it contains multiple genes encoding for the spliceosome sub-units 2 and 5. Alternative splicing has been recently implicated as a component of host-pathogen interactions in human and other mammals (Boudreault et al., 2019; Chauhan et al., 2019; Rotival et al., 2019; Yamaguchi et al., 2022; Lyu et al., 2023), including direct interactions between viral proteins and sub-unit 5 (U5) (Boudreault et al., 2022a; Boudreault et al., 2022b). Thus, it is plausible that variants of the spliceosome subunits may have an impact on the host-pathogen interactions after IHNV infection, and potentially may be the causative variant or variants for resistance to IHNV infection that was detected on chromosome Y QTL.

## Conclusion

The genetic architecture of resistance to IHNV in two commercial rainbow trout aquaculture strains was found to be oligogenic. None of the QTL detected had a large enough effect on the genetic variance for the trait that would merit marker assisted selection. Four of the five most supported QTL contain genes that may encode proteins that can be involved in antiviral function or that regulate components of the innate or adaptive immune response of the host. The fifth contain multiple sub-units of the spliceosome, which has also been recently implicated to be involved in host-pathogen interactions in human and other mammals. Further evaluation to refine those QTL regions with more genetic markers will bring us closer to the identification of the causative genome variants that are associated with IHNV resistance in the two commercially important rainbow trout aquaculture strains.

## Data Availability

The datasets presented in this study can be found in online repositories. The names of the repository/repositories and accession number(s) can be found in the article/Supplementary Material.

## Supplementary material

SUPPLEMENTARY FILE S1 Perl script to assign best trio matching of parental pair genotypes to offspring genotypes.

SUPPLEMENTARY FILE S2 Map of markers’ genome positions for TLUM 2019.

SUPPLEMENTARY FILE S3 Map of markers’ genome positions for TLUN 2018.

SUPPLEMENTARY FILE S4 Genes located within the most supportive 1-Mb QTL windows.

SUPPLEMENTARY FILE S5 GO terms assigned to the genes in the QTL windows.

SUPPLEMENTARY FILE S6 Overlapping QTL regions with the Clear Springs Food study (Vallejo et al., 2019).
