# Supplementary material for: Genome-wide association analysis of the resistance to infectious hematopoietic necrosis virus in two rainbow trout aquaculture lines confirms oligogenic architecture with several moderate effect quantitative trait loci
Source: Front Genet. 2024 May 24;15:1394656. doi: 10.3389/fgene.2024.1394656 (PMC11162110; doi:10.3389/fgene.2024.1394656)
Supplement: Supplementary file 3 [file Table1.DOCX]

#!/usr/bin/perl -w

use strict;

my $fsm = 'My_IHNV_Challenge_Nov2018_Samples.txt';

open(FSM, $fsm) or die;

<FSM>;

my %p2u = ();

my %u2p = ();

my $nfish = 0;

my %fish = ();

while(<FSM>) {

chomp;

my ($p, $u) = split /\t/;

unless($u eq '2013_399_918') {

$p2u{$p} = $u;

$u2p{$u} = $p;

++$nfish;

$fish{$u} = 1;

}

}

print "Found $nfish fish samples.\n";

my $fpd = 'Parents_with_genotype.txt';

open(FPD, $fpd) or die;

<FPD>;

my @fam = ();

my %sire = ();

my %dam = ();

my $nfam = 0;

while(<FPD>) {

chomp;

my ($f, $s, $d) = split /\t/;

unless(defined($fish{$s}) and defined($fish{$d})) {

print "check $f parents $d $d.\n";

}

unless(defined($sire{$f})) {

push @fam, $f;

$sire{$f} = $s;

$dam{$f} = $d;

++$nfam;

$fish{$s} = 2;

$fish{$d} = 2;

}

}

print "Will check with $nfam families.\n";

my @prog = ();

my $nprog = 0;

foreach my $fi (keys %fish) {

unless ($fish{$fi} == 2) {

push @prog, $fi;

++$nprog;

}

}

print "Will check for $nprog offspring fish.\n";

my $fph = '../SNPolisher_out_default/Recommended.ps';

open(FPH, $fph) or die;

my %isph = ();

<FPH>;

my $nph = 0;

while(<FPH>) {

chomp;

$isph{$_} = 1;

++$nph;

}

print "Check with $nph PolyHigh markers.\n";

my $in = '../My_IHNV_Challenge_Nov2018_CallCode.txt';

open(IN, $in) or die;

chomp(my $hed = <IN>);

my @pid = split /\t/, $hed;

shift @pid;

my %fgt = ();

my @mrk = ();

my $nm = 0;

while(<IN>) {

chomp;

my @tmp = split /\t/;

my $m = shift @tmp;

if (defined($isph{$m})) {

++$nm;

push @mrk, $m;

for my $i (0..$#tmp) {

unless($tmp[$i] eq 'NoCall') {

my $p = $pid[$i];

if (defined($p2u{$p})) {

my $u = $p2u{$p};

$fgt{$m}->{$u} = $tmp[$i];

}

}

}

}

}

close(IN);

print "Found $nm markers in the genotype file.\n";

my $out = 'assign_parents_chk.txt';

open(OUT, ">$out") or die;

print OUT "Progeny\tFamily\tSire\tDam\tTotal_markers\tWrong_geno\tR_wrong\n";

my %minr = ();

my %mint = ();

my %minf = ();

my %fsel = ();

foreach my $p (@prog) {

foreach my $f (@fam) {

my $s = $sire{$f};

my $d = $dam{$f};

my $nf = 0;

my $tot = 0;

foreach my $m (@mrk) {

if (defined($fgt{$m}->{$s}) and defined($fgt{$m}->{$d})

and defined($fgt{$m}->{$p})) {

++$tot;

my $gprog = $fgt{$m}->{$p};

my $gdam = $fgt{$m}->{$d};

my $gsire = $fgt{$m}->{$s};

my $chk = pedcheck($gprog, $gdam, $gsire);

if ($chk == 0) {

++$nf;

last if ($nf > 6000);

}

}

}

if ($tot>0) {

my $r = $nf / $tot;

if ($r < 0.1) {

print OUT "$p\t$f\t$s\t$d\t$tot\t$nf\t$r\n";

print "$p\t$f\t$s\t$d\t$tot\t$nf\t$r\n";

unless(defined($minr{$p})) {

$minr{$p} = $r;

$mint{$p} = $tot;

$minf{$p} = $nf;

$fsel{$p} = $f;

} elsif($r < $minr{$p}) {

$minr{$p} = $r;

$mint{$p} = $tot;

$minf{$p} = $nf;

$fsel{$p} = $f;

}

}

}

}

}

my $fsl = 'assign_parents_sel.txt';

open(FSL, ">$fsl") or die;

print FSL "Progeny\tFamily\tSire\tDam\tTotal_markers\tWrong_geno\tR_wrong\n";

foreach my $p (keys %minr) {

my $f = $fsel{$p};

my $d = $dam{$f};

my $s = $sire{$f};

my $tot = $mint{$p};

my $nf = $minf{$p};

my $r = $minr{$p};

print FSL "$p\t$f\t$s\t$d\t$tot\t$nf\t$r\n";

}

exit;

sub pedcheck {

my ($gprog, $gdam, $gsire) = @_;

my $chk = 1;

if ($gdam eq $gsire and ($gsire eq 'AA' or $gsire eq 'BB')) { # monomorphic

if ($gprog eq 'AB') {

$chk = 0;

}

} elsif (($gdam eq 'AA' and $gsire eq 'BB') or ($gdam eq 'BB' and $gsire eq 'AA')) { # AA/BB

if ($gprog ne 'AB') {

$chk = 0;

}

} elsif (($gdam eq 'AB' and $gsire eq 'AA') or ($gdam eq 'AA' and $gsire eq 'AB')) { # AB/AA

if ($gprog eq 'BB') {

$chk = 0;

}

} elsif (($gdam eq 'AB' and $gsire eq 'BB') or ($gdam eq 'BB' and $gsire eq 'AB')) { # AB/BB

if ($gprog eq 'AA') {

$chk = 0;

}

} else {

if ($gdam ne 'AB' or $gsire ne 'AB') {

print "Please check the genotype $gprog, $gdam, $gsire.\n";

exit;

}

}

return $chk;

}
